# Flexibles Gleichungslösen im Klassengespräch unterstützen – der Beitrag des Vergleichens von multiplen Lösungswegen

**DOI:** 10.1007/s13138-023-00221-5

**Published:** 2023-05-04

**Authors:** Christian Serop Hämmerle

**Affiliations:** Institut für Erziehungswissenschaft, Universität Zürich, Zürich, Schweiz

**Keywords:** Gleichungslösen, Klassengespräch, Vergleichen, Flexibilität, Sekundarstufe, Equation solving, Classroom discourse, Comparing, Flexibility, Secondary school, C30, C50, C60, C70, D30, D50, H20, H30, Q30, Q40

## Abstract

Damit Lernende Gleichungen zielorientiert lösen können, sind Planungsprozesse notwendig. Planungsprozesse schließen die Evaluation von möglichen Lösungswegen mit ein und werden von Ausführungs- und Strukturierungsprozessen abgegrenzt. Das Planen von geeigneten Lösungswegen wird in der Literatur mit dem Begriff der Flexibilität verknüpft. Vergleiche von multiplen Lösungswegen haben sich für die Flexibilität als lernförderlich erwiesen. Um die Lernförderlichkeit der Vergleiche zu unterstützen, werden produktive Klassengespräche empfohlen, in denen die Gegenüberstellung der Lösungswege besprochen wird. Dieser Beitrag prüft, ob in Klassengesprächen zu Vergleichen von multiplen Lösungswegen Planungsprozesse häufiger thematisiert werden als beim Besprechen nur eines Lösungsweges und als beim Besprechen von multiplen Lösungswegen ohne Vergleich. Die Stichprobe der Inhaltsanalyse umfasst Klassengespräche aus 172 Lektionen und 43 Klassen (Jahrgangsstufe 9 und 10). Die statistische Analyse wird sowohl klassenübergreifend mit binär logistischen Regressionsmodellen durchgeführt als auch klassenspezifisch mit t‑Tests für paarweise verbundene Stichproben. Die Studie zeigt, dass beim Vergleichen von multiplen Lösungswegen etwa doppelt so häufig Planungsprozesse thematisiert werden. Zusätzlich wird dokumentiert, dass beim Lösen von Gleichungen Ausführungsprozesse am häufigsten besprochen werden.

## Abstract (en)

Students struggle with planning suitable solution methods in equation solving. Planning suitable solution methods is key to flexibility, a desired skill for equation solving. Comparing solution methods has been shown to foster flexibility. To support the learning benefits of the comparisons, productive classroom talk, which includes the discussion of different solution methods, is recommended. This study examines whether discussions that compare multiple methods include more planning processes than discussions that do not compare multiple solution methods or that are just about one solution method. The content analysis is based on utterances from 172 lessons from 43 classrooms in grades 9 and 10. The hypothesis is tested both *across classes* using binary logistic regression models and *at the class level* using paired samples t‑tests. The results show that planning processes are addressed about twice as often when comparing multiple solution methods. Additionally, the study finds that enacting solution methods is the most frequent topic in classroom talk about solving equations.

## Einleitung

Viele Schülerinnen und Schüler haben Mühe mit dem Lösen von Gleichungen (vgl. Malle 1993; Rüede 2015; Thorndike et al. 1926; Van Stiphout 2011). Die Schwierigkeiten mit diesem Kernthema der Sekundarstufen 1 und 2 deuten darauf hin, dass das Automatisieren beim Lösen von Gleichungen zu wenig mit dem Verständnisaufbau einhergeht (Freudenthal 1983). Ein Beispiel: Von 134 Lernenden (gymnasiale Jahrgangsstufen 9 und 10) lösen ca. 90 % die Gleichung *x* − *x* (3*x* + 4) = 5 − *x* (3*x* + 4), indem sie im ersten Schritt ausmultiplizieren. Ein Drittel erhält mit diesem Verfahren ein falsches Resultat (Hämmerle et al. 2018). Für geübte Augen ist die Lösung *x* = 5 hingegen offensichtlich und lässt sich formal mit der Addition von *x* (3*x* + 4) auf beiden Seiten der Gleichung herleiten. 90 % der Lernenden sind demnach nicht in der Lage, für diese Gleichung einen geeigneten Lösungsweg zu finden.

Um diese Schwierigkeiten der Lernenden anzugehen, braucht es eine klare Vorstellung des Gleichungslöseprozesses. Im vorliegenden Artikel wird das Lösen von Gleichungen als ein Problemlösen verstanden (Schoenfeld 1987) und in drei Prozesse gegliedert: Strukturieren, Planen und Ausführen (Malle 1993; Rüede und Staub 2019). Planen umfasst bspw. das Erwägen von möglichen Lösungswegen und deren Evaluation. Der Planungsprozess ist kritisch für das Erkennen und Auswählen eines geeigneten Lösungsweges. Im obigen Beispiel scheitern 90 % der Lernenden beim Planen eines geeigneten Lösungswegs. Das Auswählen eines geeigneten Lösungsweges und die anschließende Berechnung der Lösung wird in der Literatur als flexibles Gleichungslösen verstanden (Verschaffel et al. 2011, S. 192f.). Weil die Flexibilität für das Lösen von Gleichungen wesentlich ist, wird sie auch curricular gefordert (vgl. National Council of Teachers of Mathematics 2006; Rüede et al. 2019). Als didaktischer Zugang zur Förderung der Flexibilität hat sich das Vergleichen von Lösungswegen etabliert (vgl. Forschungsarbeiten von Rittle-Johnson und Star; für einen Überblick Durkin et al. 2017). In solchen Vergleichsaufgaben werden jeweils zwei ausgearbeitete und korrekte Lösungswege zu einer Gleichung gegenübergestellt. Die Gegenüberstellung lenkt die Aufmerksamkeit auf Planungsprozesse, etwa die Evaluation der Lösungswege, indem zum Beispiel bedeutsame Unterschiede herausgearbeitet werden. Klassengespräche (manchenorts auch Unterrichtsgespräche oder Klassendiskussionen genannt) sind eine geeignete Sozialform, um die Lernwirksamkeit der Vergleichsaufgaben zu unterstützen (Lynch und Star 2014; Durkin et al. 2021). Das lässt vermuten, dass Planungsprozesse in diesen Klassengesprächen zu Vergleichen vermehrt besprochen werden. Diese Vermutung wird im vorliegenden Artikel empirisch untersucht, indem Klassengespräche zum Lösen von Gleichungen mit Bezug auf die thematisierten Gleichungslöseprozesse analysiert werden.

Das Datenmaterial dieses Beitrags entstand im Rahmen des Schweizer Nationalfondsprojekts MathFlex^1^ „Förderung von algebraischer Flexibilität. Wirkungen von Weiterbildungen zum Vergleichen von Lösungswegen im gymnasialen Mathematikunterricht“ (Rüede et al. 2023). In dieser Interventionsstudie mit experimentellem Design besuchten die 30 Lehrpersonen der Experimentalgruppen eine Weiterbildung zum Vergleichen von Lösungswegen im Klassengespräch. An der viertägigen Weiterbildung wurden den Lehrpersonen Vergleichsaufgaben zu quadratischen Gleichungen abgegeben. Weitere 13 Lehrpersonen zählten zur Wartekontrollgruppe. Alle Lehrpersonen unterrichteten mit ihrer Klasse das Thema der quadratischen Gleichungen in 16 Lektionen. Das deklarierte Lernziel war „quadratische Gleichungen flexibel lösen können und verstehen, was man dabei macht“. Pro Klasse wurden vier Lektionen zum Lösen quadratischer Gleichungen videografiert (insgesamt 172). Die vorliegende Untersuchung nutzt diese Videografien als Gelegenheitsstichprobe zur Untersuchung der gestellten Forschungsfrage. Im Unterschied zu anderen Analysen innerhalb des Projekts erfolgt im vorliegenden Beitrag eine Auswertung der Klassengespräche, welche sich auf das Besprechen von Gleichungslöseprozessen bezieht.

## Theorie

### Strukturieren, Planen und Ausführen – drei Prozesse beim Lösen von Gleichungen

Im Fokus dieses Artikels steht das Lösen von Gleichungen mit Hilfe von algebraischen Umformungen. Typisch für den Schulunterricht werden Gleichungen mit nur einer Variablen betrachtet, was sich aber theoretisch erweitern lässt. Das Ziel des Lösens ist es, einen Zahlenwert für die Variable zu finden, so dass die Gleichung eine wahre Aussage ergibt. Damit entspricht das Gleichungslösen einem Problemlösen, bei dem ein Anfangszustand über eine Transformation in einen Endzustand gebracht wird (Schwarz 2018). Die Perspektive des Problemlösens erlaubt es, die Schwierigkeiten der Lernenden im Lösungsprozess besser zu beschreiben.

Polya (1945) und Schoenfeld (1987) haben wichtige Beiträge zum Problemlösen im Mathematikunterricht geleistet. Sie gliedern das Problemlösen in ein Verstehen des Problems, Planen des Lösungsweges, Ausführen des Plans und Zurückschauen. Diese Gliederung entspricht nicht zwingend einer Chronologie: Schoenfeld (1985, Fig. 4.2, S. 110) weist auf mögliche zirkuläre Verläufe hin. In einem zirkulären Verlauf beeinflussen sich die Prozesse gegenseitig, so dass zum Beispiel das Zurückschauen nach einem Schritt in ein neuerliches Planen übergeht. Diese gegenseitige Beeinflussung der Prozesse nimmt auch Malle in seine Beschreibung des Gleichungslösens auf (Malle 1993, Fig. 72, S. 190). Er unterteilt sein dreiseitiges Modell in die Prozesse Termstrukturen erkennen, Anwenden von Regeln, heuristische Strategien. Rüede und Staub (2019) sowie der vorliegende Artikel lehnen sich mit den Begriffen Strukturieren, Planen und Ausführen an diese Dreiteilung an. Nachfolgend werden diese drei Prozesse anhand der Gleichung (*x* + 1)^2^ = 49 erläutert.

Beim Lösen einer Gleichung müssen zuerst die mathematischen Zeichen interpretiert werden (vgl. Malle 1993; Rüede 2015). Dieser Interpretationsprozess wird als *Strukturieren* bezeichnet und meint, dass mögliche Strukturen erkannt werden. Dazu gehört bspw. zu erkennen, dass es sich um eine Gleichung handelt, dass auf beiden Seiten der Gleichung ein Quadrat steht oder dass 49 eine natürliche Zahl ist. Der Strukturierungsprozess verbindet die Zeichen und weist ihnen eine Bedeutung zu.

Das *Ausführen* bezieht sich sowohl auf einzelne Term- und Äquivalenzumformungsregeln (wie das Ausmultiplizieren von (*x* + 1)^2^) als auch auf ganze Verfahren. Ein Lösungsverfahren für die quadratische Gleichung (*x* + 1)^2^ = 49 ist das Umformen auf die allgemeine Form und das darauffolgende Anwenden einer Lösungsformel. Auch das Rechnen mit Zahlen entspricht solchen Ausführungsprozessen.

In dieser Lesart bestimmen Strukturierungsprozesse, welche Regel ausgeführt wird. Weil Strukturen und Regeln aber nicht unabhängig voneinander sind, lässt sich umgekehrt argumentieren, dass Regeln ihrerseits bestimmen, wie strukturiert wird. Am Beispiel (*x* + 1)^2^ lässt sich darüber streiten, ob der Term eher als Produkt gedeutet wird (und damit eine Strukturierung darstellt) oder als Term mit der Eigenschaft, dass er ausmultipliziert werden kann. So beeinflussen sich die verschiedenen Gleichungslöseprozesse gegenseitig.

Damit die Strukturierungs- und Ausführungsprozesse sinnvoll verknüpft werden können, braucht es das *Planen*. Es beinhaltet die zielorientierte Suche nach einem Lösungsweg und dessen Evaluation (Rüede und Staub 2019). Um das „Wann“ (nach Sfard 2008) einer Umformung oder einer Abfolge von Umformungen einschätzen zu können, müssen verschiedene Kriterien berücksichtigt werden: die Ausgangslage des Problems, das Ziel der Problemstellung, die Effizienz, Eleganz und Generalisierbarkeit eines Lösungsweges und zusätzlich dessen subjektive Bewertung zum Beispiel hinsichtlich der Fehleranfälligkeit. Um die Gleichung (*x* + 1)^2^ = 49 zielorientiert zu lösen, lässt sich die quadratische Struktur links und rechts der Gleichung elegant ausnutzen – ohne das Ausmultiplizieren des Klammerterms. Beim eleganteren Weg gilt es darauf zu achten, dass die negative Lösung beim Radizieren nicht vergessen wird. Solche kognitiven Prozesse werden vorliegend als Planungsprozesse bezeichnet. Das führt zu einem weiten Begriff des Planens und zu einer Verbindung mit metakognitiven Prozessen beim Lösen von Gleichungen (Garofalo und Lester 1985). Desoete (2008) und Lucangeli, Cornoldi und Tellarini (1998) unterscheiden für die Mathematik vier wichtige metakognitive Prozesse (engl. *skills*): *prediction, planning, monitoring, evaluation*. Die metakognitiven Prozesse beschreiben das vorgängige Planen (inkl. *prediction*) der Lösung eines Problems, das Überwachen der Problemlösung und die Evaluation der Problemlösung. Im zirkulären Modell von Schoenfeld (1985) bzw. im dreiseitigen Modell von Malle (1993) können diese metakognitiven Prozesse beim Gleichungslösen prinzipiell immer stattfinden. Zudem werden beim Planen, Monitoring und Evaluieren dieselben Kriterien miteinbezogen: Die Effizienz oder Eleganz eines Lösungsweges genauso wie die subjektive Einschätzung des Weges (z. B. bezüglich der Fehleranfälligkeit). Für den Kompetenzaufbau lässt sich deshalb das Planen, Monitoring und Evaluieren sinnvoll zusammenfassen. Im vorgelegten Artikel umfassen Planungsprozesse neben dem eigentlichen Planen also auch Monitoring- und Evaluationsprozesse.

### Flexibles Gleichungslösen und das Planen

Weil beim Planen mögliche Lösungswege evaluiert werden müssen, kommt dem Planen eine wichtige Funktion beim Auswählen eines Lösungsweges zu. Dieser Auswahlprozess wird in der Literatur mit dem Begriff der Flexibilität verknüpft: Das Auswählen (engl*. selection*) des geeignetsten Lösungsweges (innerhalb eines gegebenen Kontexts) und Berechnen der Lösung (engl.* execution*) wird als flexibles Gleichungslösen verstanden (Verschaffel et al. 2011, S. 192f.; ähnlich unter dem Begriff der prozeduralen Flexibilität bei Rittle-Johnson et al. 2012). Die Kompetenz der Flexibilität wird in Lehrplänen und Unterrichtsempfehlungen gefordert (z. B. in den deutschen Bildungsstandards im Fach Mathematik für die allgemeine Hochschulreife, KMK 2012; im Anhang zum schweizerischen Rahmenlehrplan für Maturitätsschulen der EDK (2016); in den USA z. B. durch Swafford und Findell 2001). Die Wichtigkeit der Flexibilität für den erweiterten Wissenserwerb lässt sich lerntheoretisch begründen (Spiro 1988), was Star und Rittle-Johnson (2008) für das Lösen von Gleichungen verdeutlichen. Folgend wird das Planen mit dem flexiblen Gleichungslösen genauer in Beziehung gesetzt.

Das Auswählen eines geeigneten Lösungsweges bedeutet an der Gleichung (*x* + 1)^2^ = 49, dass mindestens zwei Lösungswege mental angedacht werden, zum Beispiel ein Lösungsweg über das Ausmultiplizieren des Klammerterms und ein zweiter Lösungsweg über das Ziehen der Wurzel auf beiden Seiten der Gleichung. Im Auswahlprozess werden die Wege evaluiert, indem zum Beispiel die Effizienz als Kriterium beigezogen wird. Weil der zweite Lösungsweg weniger Rechenschritte benötigt, ist er effizienter und darum geeigneter und also flexibler. Flexibilität heißt deshalb in diesem Fall auch, dass mathematische Symbole, wie der quadrierte Klammerterm, nicht als Rechenanweisungen gelesen werden (vgl. Nührenbörger und Schwarzkopf 2015; Sfard 2008). Mangelnde Flexibilität wäre demnach Ausdruck von defizitären Planungsprozessen. Darum muss für das zielorientierte (d. h. flexible) Lösen von Gleichungen das Planen aufgebaut werden.

### Das Vergleichen von Lösungswegen zur Förderung der Flexibilität

**Figure 1:**
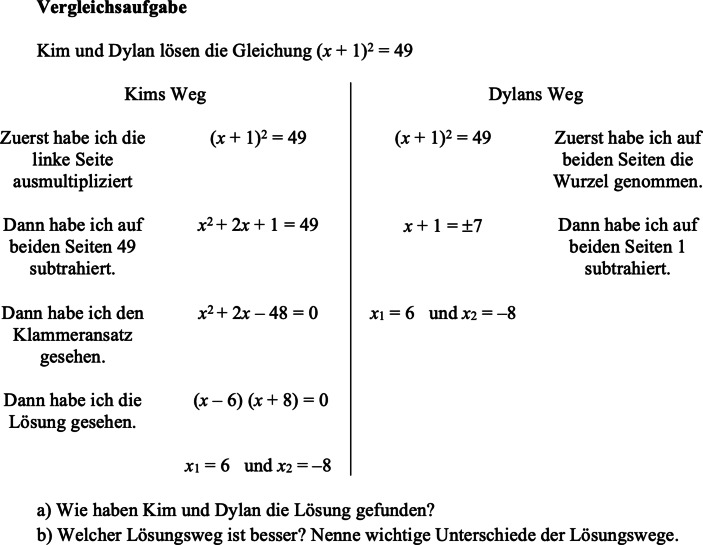

Zur Förderung des flexiblen Gleichungslösens stellt das Vergleichen von multiplen Lösungswegen einen wichtigen didaktischen Zugang dar. Allgemein ist das Vergleichen und Kontrastieren wichtig zur Wissensvertiefung (z. B. Silver et al. 2005; Schukajlow et al. 2015; Lipowsky und Hess 2019; Lipowsky et al. 2019). In experimentellen Laborstudien konnte im Bereich der Algebra gezeigt werden, dass das flexible Gleichungslösen von Lernenden durch das Vergleichen von multiplen Lösungswegen gefördert werden kann (für einen Überblick Durkin et al. 2017). In diesen Studien werden Vergleichsaufgaben genutzt, die jeweils zu genau einer Gleichung zwei korrekte Lösungswege als sogenannte *worked examples* zeigen (vgl. Abb. 1 nach Durkin et al. 2021; oder auch Rüede und Staub 2019). Lernende sollen diese Wege studieren und danach zugehörige Teilaufgaben bearbeiten. Die Teilaufgaben zum Vergleich der Lösungswege zielen darauf ab, zuerst die Korrektheit der Lösungswege nachzuvollziehen und danach die Lösungswege auf Gemeinsamkeiten und Unterschiede zu evaluieren. Die erste Frage „Wie haben Kim und Dylan die Lösung gefunden?“ kann somit einerseits auf die vorliegenden Ausführungsprozesse bezogen werden, indem zum Beispiel versucht wird nachzuvollziehen, wie Kim im letzten Schritt die Lösungen erhält. Andererseits kann verdeutlicht werden, dass zweimal der Term (*x* + 1)^2^ korrekt interpretiert wird, dass aber nur Dylan die beidseitige quadratische Struktur nutzt. Die zweite Teilaufgabe fordert eine Evaluation der Lösungswege („Welcher Weg ist besser?“) und das Nennen von Unterschieden. Solche Vergleichsaufgaben thematisieren demnach Planungsprozesse, indem durch die Evaluation verschiedene Strukturierungen einer Gleichung mit den verschiedenen Ausführungsprozessen verbunden werden.

In der Forschungsliteratur treten manchmal statt des Vergleichs von multiplen Lösungswegen die multiplen Lösungswege selbst ins Zentrum (z. B. Große 2014; Achmetli et al. 2019; Leikin 2011). Es ist jedoch zu vermuten, dass zur Förderung der Flexibilität Lösungswege miteinander verglichen werden sollten. Denn multiple Lösungswege ohne einen Vergleich fokussieren nicht notwendigerweise auf die Planungsprozesse. In diesem Sinn könnten auch die Ergebnisse von Krug und Schukajlow (2020) gedeutet werden, wo die Aufforderung an die Lernenden, selbst multiple Lösungswege zu generieren, zwar zu häufigeren Planungsprozessen (dort als prozedurale Metakognition bezeichnet) führte, aber die Ergebnisse teilweise an der Signifikanzgrenze lagen. So finden Vergleiche nicht unbedingt statt, wenn sie nicht angeleitet werden. Rittle-Johnson und Star (2007) konnten zudem aufzeigen, dass das sequenzielle Bearbeiten multipler Lösungswege für die Lernenden weniger lernwirksam für die Ausbildung von Flexibilität ist als das parallele Bearbeiten von multiplen Lösungswegen. Darum stehen die Lösungswege bei solchen Vergleichsaufgaben (wie in Abb. 1) nebeneinander und regen dadurch den Vergleich stärker an. Um diesen Vergleich im Unterricht noch besser herauszuarbeiten, sind Klassengespräche von grundlegender Bedeutung (vgl. Durkin et al. 2021).

### Produktive Klassengespräche

Klassengespräche sind nicht nur für Vergleichsaufgaben eine wichtige Sozialform. Sie eignen sich allgemein für den Wissensaufbau im Unterricht (Stigler et al. 1999). Darum nahm in den letzten Jahren die erziehungswissenschaftliche Unterrichtsforschung Klassengespräche vermehrt als Ausgangspunkt (als Überblick Mercer und Dawes 2014; für neuere Forschungsbeiträge z. B. Resnick et al. 2015; aus mathematikdidaktischer Perspektive Herbel-Eisenmann et al. 2017). Aus sozialkonstruktivistischer Perspektive (nach Vygotsky z. B. 1978) wird Wissen im Klassengespräch nicht „vermittelt“, sondern durch den sozialen Diskurs (im Unterricht) dialogisch konstruiert. Die Lehrperson unterstützt dabei gezielt die gemeinsame Konstruktion in sogenannt produktiven Klassengesprächen, zum Beispiel mit spezifischen Gesprächshandlungen (sog. „talk moves“; Michaels et al. 2010; Mercer und Howe 2012; Pauli und Reusser 2018). Dadurch sollen Lernende angeregt werden, ihre Gedanken explizit zu machen, den anderen zuzuhören, eigene sowie Argumente von anderen weiterzuentwickeln und zu hinterfragen. Solche Gesprächshandlungen können Äußerungen sein wie „Was denkst du?“, „Kann jemand etwas dazu anfügen?“ oder „Warum stimmt diese Bemerkung?“.

Die genannten Analysen und der Fokus auf die Gesprächshandlungen konnten aufzeigen, wie Klassengespräche produktiv gestaltet werden können (s. a. Howe et al. 2019). Die Produktivität der Klassengespräche wird auf die kommunikativen Funktionen von Äußerungen im Klassengespräch bezogen (s. a. Boston 2012; Hennessy et al. 2020; Pehmer et al. 2014; Drageset 2015). Das heißt, die Analysen beziehen sich viel mehr darauf, wie (!) kommuniziert werden soll, als darauf, was kommuniziert werden soll. Als Beispiel kann ein gängiges Instrument zur Klassengesprächsanalyse dienen: das Instructional Quality Assessement (IQA, Boston 2012). Im IQA gelten Mathematiklektionen dann als die gehaltvollsten, wenn „die Fragen einer Lehrperson durchgehend akademisch relevant sind und den Schülerinnen und Schülern Gelegenheit bieten, ihr mathematisches Arbeiten und Denken weiterzuentwickeln und zu erklären“ (deutsche Übersetzung aus Boston 2012, S. 11). Eine fachliche Komponente in Form der „akademischen Relevanz“ oder des „mathematischen Arbeitens“ wird zwar berücksichtigt, aber nicht genauer beschrieben. Dass für die Beurteilung der Unterrichtsqualität eine fachliche Ausrichtung der Klassengesprächsanalysen vermehrt berücksichtigt werden sollte, legen neueste Studien dar (Jentsch et al. 2020; Praetorius und Charalambous 2018; Schlesinger und Jentsch 2016).

Die sozialkonstruktivistische Gesprächsanalyse fand auch Eingang in die Mathematikdidaktik. Nührenbörger (2009) zeigte bspw. aus einer epistemischen Perspektive das Spektrum an Analysedimensionen auf, die beim gemeinsamen Bearbeiten von mathematischen Aufgaben durch Lernende entstehen. Im Kontext von Vergleichen stellen Kooloos und sein Forschungsteam (Kooloos et al. 2020) anhand von analysierten Klassengesprächen fest, dass der Einsatz von multiplen Lösungswegen in der analytischen Geometrie die Klassengespräche insofern veränderte, als die Lösungswege selbst thematisiert wurden, sich mehr Lernende äußerten und der Unterricht offener wurde. Für die Untersuchung von Klassengesprächen in Verbindung mit den mathematischen Lernzielen des Unterrichts stellt Drollinger-Vetter (2011) einen wichtigen Bezugspunkt dar. Ihre Arbeit findet sich im Umfeld der schweizerisch-deutschen Videostudie zur Satzgruppe des Pythagoras, welche Unterrichtsqualität, Lernverhalten und mathematisches Verständnis untersuchte (für einen Überblick vgl. Reusser und Pauli 2013). Drollinger-Vetter (2011) analysiert Klassengespräche innerhalb des Inhaltsbereichs der Satzgruppe des Pythagoras, indem sie sogenannte Verstehenselemente bestimmt. Diese Verstehenselemente beschreiben die mathematischen Inhalte, welche für ein vertieftes konzeptuelles Wissen zum Satz des Pythagoras notwendig sind, wie den Geltungsbereich des Satzes oder den Unterschied zwischen Kathete und Hypotenuse. So konnten bspw. Unterrichtsphasen danach untersucht werden, ob diese Verstehenselemente häufig, selten oder gar nicht vorkommen. Die fachliche Qualität (gemessen am Vorkommen der Verstehenselemente, an der Qualität der Repräsentation und an der strukturellen Klarheit des Unterrichts) stellte bei der Satzgruppe des Pythagoras den besten Prädiktor des Lernerfolgs der Lernenden dar (vgl. Reusser und Pauli 2013, S. 319). Das zeigt, wie wichtig die fachliche Auseinandersetzung mit den Lernzielen in den Klassengesprächen für das Lernen der Schülerinnen und Schüler ist. Für eine Klassengesprächsanalyse zum Aufbau des flexiblen Gleichungslösens müssen diese Verstehenselemente für das Gleichungslösen erweitert werden. Als Erweiterung bietet sich die Beschreibung des Gleichungslösens durch Strukturieren, Planen und Ausführen an.

### Das Thematisieren von Planungsprozessen in Klassengesprächen

Im Klassengespräch zum Lösen von Gleichungen wird über die Prozesse des Strukturierens, Planens und Ausführens gesprochen. Dieses Sprechen dient dem gemeinsamen Kompetenzaufbau. Folgende Äußerung thematisiert bspw. einen Ausführungsprozess beim Lösen der Gleichung (*x* + 1)^2^ = 49: „Und dann ziehe ich die Wurzel. Das gibt *x* plus 1 gleich 7, äh, ich meine, plus-minus 7.“ Diese Äußerung lässt sich nicht nur als ein Berichten eines Schrittes interpretieren, sondern auch als ein Ausführen des Schrittes – unabhängig davon, ob dieser Schritt ad hoc entsteht oder aufgrund eines vorgelösten Lösungsweges berichtet wird. Genauso können die Zuhörenden mit Hilfe der genannten Äußerung diesen Schritt (mental) ausführen. Weitere Beispiele zur Thematisierung von Ausführungsprozessen sind „Zuerst rechne ich minus 49.“, „Darf ich einfach die Wurzel ziehen?“ oder „Wende das Standardverfahren an!“. Das Besprechen von Strukturierungsprozessen akzentuiert die strukturelle Interpretation von Termen und Gleichungen: „Warum ist (*x* + 1)^2^ = 49 eine quadratische Gleichung?“; „Die Variable ist *x*.“; „Auf beiden Seiten der Gleichung steht ein Quadrat.“. Neben Ausführungs- und Strukturierungsprozessen werden auch Planungsprozesse gemeinsam besprochen: „Um die Parameter *a, b* und *c* der quadratischen Gleichung herauszufinden, muss man zuerst ausmultiplizieren.“ oder „Die Quadrate auf beiden Seiten der Gleichung lassen sich zur Lösung der Gleichung ausnutzen.“. In beiden Fällen werden Strukturen (Parameter bzw. Quadrate) mit Ausführungsschritten (Ausmultiplizieren, „zur Lösung nutzen“) zielorientiert verbunden. Daher sind es Planungsprozesse (vgl. Abschn. 2.1).

Bauersfeld (1992) legte dar, dass eine Diskussion zu Vergleichen (dort als *solved tasks *und* contrasting *bezeichnet) eine flexible Interpretation von mathematischen Objekten möglich macht, wenn dabei der Fokus auf die gemeinsame Wissenskonstruktion der Lernenden gelegt wird. Für die Förderung des flexiblen Gleichungslösens müssten beim Vergleichen von Lösungswegen Planungsprozesse vermehrt gemeinsam besprochen werden (vgl. Abschn. 2.2). Denn für den fachlichen Kompetenzaufbau ist wesentlich, was im Klassengespräch während des Vergleichens von Lösungswegen thematisiert wird und inwiefern die Lerngelegenheit des Vergleichs für das Planen genutzt wird. Anhand der ersten Teilaufgabe von Abb. 1 im Klassengespräch könnte nicht nur diskutiert werden, „wie“ Dylan die Lösung berechnet hat, sondern auch, warum genau diese Strategie gewählt wurde (z. B. um keine quadratische Gleichung lösen zu müssen). So werden schon vor dem Vergleich der beiden Lösungswege Planungsprozesse besprochen. Entscheidend ist, dass dieser Diskussionspunkt erst durch die Präsenz der zwei Lösungswege zum Gegenstand des Interesses wird. Durch die (abgebildeten) Alternativen wird offensichtlich, dass die Gleichung verschieden strukturiert werden kann. Zudem lassen sich die abgebildeten Lösungswege einfacher hinsichtlich Effizienz oder Ästhetik evaluieren, weil zum Beispiel Rechenschritte gezählt werden können.

Wird statt des Vergleichs von zwei Lösungswegen nur ein Lösungsweg betrachtet, bieten sich entsprechend weniger Lerngelegenheiten zur Thematisierung von Planungsprozessen. Treten multiple Lösungswege auf, ohne dass diese verglichen werden, werden Planungsprozesse vermutlich nicht häufiger thematisiert, als wenn nur ein Lösungsweg gegeben ist. Damit könnte eine Verbindung zwischen dem Vergleichen von Lösungswegen und dem Entwickeln von Flexibilität über das Thematisieren von Planungsprozessen im Klassengespräch hergestellt werden.

Für Klassengespräche zum Vergleichen von multiplen Lösungswegen sind verschiedene Schwierigkeiten dokumentiert (Nathan und Knuth 2003). Silver und seine Forschungsgruppe (Silver et al. 2005) nennen zum Beispiel pädagogische und zeitliche Bedenken von Lehrpersonen. Auch Lynch und Star (2014) illustrieren, dass Lehrpersonen Mühe bekunden können, Vergleiche im Unterricht produktiv einzubringen. Durkin et al. (2021) weisen darauf hin, dass Lehrpersonen in der Orchestrierung von Klassengesprächen unterstützt werden müssen, damit Lösungswege in Klassengesprächen lernwirksam verglichen werden. Leikin (2011) zeigt, dass Lehrpersonen beim Vergleichen selbst fachlich dazu lernen können. Rüede et al. (2023) konnten zeigen, dass gezielte Lehrpersonenfortbildungen zum Vergleichen von Lösungswegen lernwirksam für die Lernenden sein können. Da in diesen Weiterbildungen das Gleichungslösen ebenfalls anhand der Prozesse des Strukturierens, Ausführens und Planens eingeführt wurde, könnte eine mögliche Ursache für die Lernzuwächse der Lernenden sein, dass die Problemlösebeschreibung anhand des Strukturierens, Ausführens und Planens den Lehrpersonen geholfen haben könnte, die Klassengespräche zu den Vergleichen fachlich zu ordnen.

### Forschungsfrage und Hypothesen

Ob Planungsprozesse beim Vergleichen von Lösungswegen vermehrt besprochen werden, wird im vorliegenden Artikel empirisch untersucht. Dafür werden die Häufigkeiten der thematisierten Gleichungslöseprozesse in Abhängigkeit von drei Besprechungsarten gesetzt. Denn Gleichungen können im Klassengespräch a) ohne multiple Lösungswege besprochen werden, b) mit multiplen Lösungswegen ohne Vergleich oder c) mit multiplen Lösungswegen mit Vergleich. Dies dient dazu besser zu verstehen, wie sich Klassengespräche zum Lösen von Gleichungen hinsichtlich dieser Prozesse voneinander unterscheiden. Unterschiede zwischen den Besprechungsarten könnten Hinweise darauf geben, dass Gleichungslöseprozesse im Unterricht unterschiedlich thematisiert werden, und erklären, warum das Vergleichen von Lösungswegen das flexible Gleichungslösen fördern kann.

#### Fragestellung:

Wie unterscheiden sich in Klassengesprächen zum Gleichungslösen die Häufigkeiten der thematisierten Gleichungslöseprozesse in Abhängigkeit von den Besprechungsarten?

Theoretisch wurde hergeleitet, dass Planungsprozesse beim Vergleichen von Lösungswegen häufiger thematisiert werden sollten, weil Evaluationen eingehender besprochen werden können. Die Häufigkeiten lassen sich dabei einerseits auf die Grundgesamtheit der Äußerungen in allen Klassengesprächen beziehen (i). So werden die Verteilungen der Gleichungslöseprozesse in Abhängigkeit der drei Besprechungsarten klassenübergreifend miteinander verglichen. Andererseits lassen sich die Häufigkeiten auch auf relative Häufigkeiten der verschiedenen Klassen beziehen (ii). So werden die relativen Verteilungen der Klassen miteinander verglichen.

#### Hypothesen:

(i) In Klassengesprächen zum Vergleichen von multiplen Lösungswegen werden Planungsprozesse *über alle Äußerungen aller Klassen hinweg* häufiger besprochen als in anderen Besprechungsarten. (klassenübergreifend)
(ii) In Klassengesprächen zum Vergleichen von multiplen Lösungswegen *einer Klasse* werden Planungsprozesse häufiger besprochen als in anderen Besprechungsarten. (klassenspezifisch)

Diese beiden Hypothesen ergänzen sich gegenseitig, weil sie gemeinsam die Verteilung der Daten adäquat miteinbeziehen. Denn ein Unterschied zwischen der Annahme und der Verwerfung der Hypothesen (i) und (ii) könnte auf eine Ungleichverteilung der Anzahl Äußerungen zurückzuführen sein. Die Ungleichverteilung könnte sowohl auf der Ebene der Klassengespräche wie auf der Ebene der Besprechungsarten zu Stande kommen, zum Beispiel wenn einzelne Klassen keine Klassengespräche führen oder nur mit einer Besprechungsart. Dass die Besprechungsart des Vergleichens nicht in allen Klassen auftritt, zeigt Star et al. (2015). Eine klassenübergreifende Perspektive (i) kann darum dokumentieren, wie Vergleiche allgemein genutzt werden. Wenn aber Klassen zum Beispiel nie Vergleichen, ist die Verteilung weniger abhängig von der Besprechungsart, sondern von der Klasse – genauso beim Besprechen der verschiedenen Gleichungslöseprozesse. Eine Berücksichtigung der Klassen (ii) ermöglicht es zudem, aus der Perspektive von Lernenden zu beurteilen, ob in einer Klasse durch die verschiedenen Besprechungsarten andere Prozesse häufiger thematisiert werden. Der Blick zwischen klassenübergreifender und klassenspezifischer Perspektive lässt so eine bessere Interpretation der durch die Klassen entstandenen Varianz zu. Darum sind beide Perspektiven einzunehmen.

## Methode

### Stichprobe

Die Stichprobe stammt aus der Interventionsstudie MathFlex (genauer beschrieben in Rüede et al. 2023; s. a. Mok et al. 2022).^2^ Die 43 teilnehmenden Lehrpersonen haben sich freiwillig zu einer viertägigen Weiterbildung zum Vergleichen von Lösungswegen in Klassengesprächen angemeldet. Die Zuteilung zu den Gruppen erfolgte randomisiert. Die Klassen der Lehrpersonen kommen aus zwölf Kantonen der Deutschschweiz und gehören zu den Schultypen Gymnasium, Fachmaturität und Berufsmaturität (9. bzw. 10. Jahrgangsstufe). In zwei Immersionsklassen wurde englisch gesprochen. Die Unterrichtssprache aller anderen Klassen war deutsch. Auch die Schülerinnen und Schüler nahmen freiwillig an der Untersuchung teil. Für 13 Lehrpersonen der Experimentalgruppe war der Fokus der Weiterbildung auf dem Vergleichen von multiplen Lösungswegen. Es wurde bspw. besprochen, inwiefern Gleichungslöseprozesse beim Vergleichen von Lösungswegen thematisiert werden können. Es wurde jedoch nicht gesagt, wann, welche oder wie oft die einzelnen Gleichungslöseprozesse thematisiert werden sollten und inwiefern diese mit Flexibilität verbunden sind. Bei den anderen 17 Lehrpersonen der Experimentalgruppe wurde zusätzlich zum Vergleichen von Lösungswegen das Führen von produktiven Klassengesprächen thematisiert. Nach diesen Weiterbildungen unterrichteten alle 30 Lehrpersonen der Experimentalgruppe das Lösen von quadratischen Gleichungen in ihren Klassen. Weitere 13 Lehrpersonen waren Teil der Wartekontrollgruppe. Sie besuchten die Weiterbildung nach dem Unterrichten der quadratischen Gleichungen. Zur Dokumentation des Unterrichts wurden vier Lektionen zu quadratischen Gleichungen videografiert, welche randomisiert aus den 16 Lektionen der Unterrichtseinheit ausgewählt wurden. Bei rund einem Drittel der Videografien lässt sich von herkömmlichem Mathematikunterricht zum Lösen von quadratischen Gleichungen sprechen, da die entsprechenden Lehrpersonen zum Zeitpunkt der Videografie noch keine Weiterbildung besucht hatten (Wartekontrollgruppe).

Im Unterschied zur Hauptuntersuchung wird vorliegend nicht zwischen den verschiedenen Gruppen unterschieden. Denn die Gruppierung steht nicht im Widerspruch zur Fragestellung mit Bezug auf die Verteilung der thematisierten Gleichungslöseprozesse in den unterschiedenen Klassengesprächen. Vor dem Hintergrund der Studie MathFlex kann aber erwartet werden, dass im videografierten Unterricht der Versuchsgruppen vermehrt multiple Lösungswege bzw. deren Vergleiche auftreten als in der Kontrollgruppe.

### Die qualitative Analyse der Klassengespräche

#### Voranalysen: Sequenzierung und Transkription der Klassengespräche

In einem ersten Analyseschritt wurden die Klassengesprächssequenzen des videografierten Unterrichts bestimmt (entsprechend Hugener 2006). Mit Hilfe der qualitativen Analysesoftware MAXQDA wurde jede Klassengesprächssequenz durch das Setzen eines Anfangs- und eines Endzeitpunkts identifiziert. Die Reliabilität der Sequenzierung wurde mit einem zweiten Rater an 5 % des Materials eingeübt und an weiteren 20 % überprüft. Mit einer gesamthaften zeitlichen Übereinstimmung von 99 % und einem Cohens κ von 0,98 zur zeitlichen Übereinstimmung ist die hohe Reliabilität auf die vielen langen Klassengespräche zurückzuführen. Die anschließende Transkription der Klassengesprächssequenzen unterteilt das Klassengespräch in aufeinanderfolgende Turns, das heißt Redebeiträge (Schegloff et al. 1974).

#### Die Codierung der thematisierten Gleichungslöseprozesse

Die Klassengesprächstranskripte wurden mit Hilfe einer qualitativen Inhaltsanalyse nach Mayring (2015) codiert. Turns der Transkripte dienten als Analyseeinheit (entsprechend Futter 2017). Da Sprechende nach Schegloff et al. (1974) etwas sagen (wollen), kann jedem Turn eine Sinneinheit zugewiesen werden. Diese Sinneinheit mit Bezug auf die thematisierten Gleichungslöseprozesse wurde allen Turns zugeordnet. Orientiert an Abschn. 2.1 werden (deduktiv) drei Gleichungslöseprozesse voneinander unterschieden: Strukturierungsprozesse (STRUKT), Ausführungsprozesse (AUSF) und Planungsprozesse (PLAN). In Tab. 1 finden sich Beispiele anhand eines transkribierten Klassengesprächs.

**Table 1:** 

#		Turn	Code	Erklärung
1	LP:	Wie erkenne ich, dass (*x* + 1)^2^ = 49 eine quadratische Gleichung ist?	STRUKT	Fragen nach einer Erklärung für eine Struktur
2	Alex:	Es steht hoch 2	STRUKT	Nennen eines Strukturmerkmals
3	Billi:	Aber *x* ist ja nicht quadriert	STRUKT	Nennen eines Strukturmerkmals
4	LP:	Ok. Andere?	CONTIN	Moderation
5	Alex:	Wenn der Klammerterm ausmultipliziert wird, steht *x* im Quadrat	PLAN	Nennen einer Struktur (Klammerterm) mit einer Umformung (ausmultiplizieren)
6	LP:	Wie berechnet ihr *x*?	AUSF	Frage nach einem Verfahren
7	Cem:	Ich mach die Klammer weg und setze dann *a, b* und *c* in die Lösungsformel ein	AUSF	Beschreiben des Verfahrens
8	LP:	Einverstanden. Das gibt sicher das richtige Resultat. Andere Lösungswege?	AUSF	Fokus auf die Korrektheit des Lösungsweges und Frage nach einem anderen Ausführungsprozess
9	Ella:	Ich habe mir überlegt, was ich bei *x* einsetzen muss, dass es dann 49 gibt. 6 wäre dann das Ergebnis	PLAN	Verbinden einer Zielstruktur (49) mit einer Ausführung (6 einsetzen)
10	LP:	Sehr schön. Gut gesehen. Einfach als Ergänzung: (−8) wäre dann auch eine Lösung, denn (−7) hoch 2 ist auch 49. Sehr gut. Welchen Lösungsweg würdet ihr hier empfehlen?	KOMB	Nennen einerseits von Ausführungsschritten (AUSF). Danach unabhängig davon das erfragen einer Evaluation (PLAN)
11	Cem:	Lösungsformel! Mir gefällt es, wenn ich einfach rechnen kann	PLAN	Evaluation
12	LP:	Mhm. Alex?	CONTIN	Moderation
13	Alex:	Ich sehe den Vorteil der Lösungsformel, aber hier finde ich den zweiten Weg einfacher	PLAN	Evaluation
14	LP:	Ok. Danke. Es klingelt schon bald wieder und ich müsste noch etwas zum Sportlager sagen …	NONGL	Kein Bezug zum Gleichungslösen

*LP* Lehrperson

Als STRUKT wurden diejenigen Turns kategorisiert, deren Thema das Nennen, Interpretieren oder Erfragen von mathematischen Strukturen (Gleichungen und Terme) beim Lösen von Gleichungen ist. „Wie haben wir diese Art von Zahl genannt?“ ist ein Beispiel einer Äußerung, welche die Interpretation einer Struktur (hier einer Zahl) in Form einer genaueren Kategorisierung („irrationale Zahl“) thematisiert (weitere Beispiele für den Code STRUKT in Tab. 1, Turn 1, 2, 3).

Als AUSF wurden diejenigen Turns kategorisiert, die das Anwenden von algebraischen Regeln, Gesetzen und Verfahren thematisieren. „Wir addieren 5 auf beiden Seiten der Gleichung.“ oder „Ich löse das mit dem quadratischen Ergänzen.“ sind Beispiele für das Thematisieren einer Regelanwendung oder eines Verfahrens (vgl. Tab. 1, Turn 6, 7). Zum Code AUSF gehört auch die Thematisierung der Korrektheit bestimmter Schritte (vgl. Tab. 1, Turn 8) und das Nennen von Ergebnissen wie „Das gibt 5.“.

Als PLAN wurden diejenigen Turns kategorisiert, welche Strukturierungsprozesse und Ausführungsprozesse zielorientiert miteinander verbinden, wie „Für die Anwendung der Auflösungsformel muss die Gleichung 0 ergeben.“. Typisch für diese Verbindung ist, wenn die Strukturierung eines Terms handlungsleitend für einen Lösungsschritt ist (Tab. 1, Turn 9) oder eine Umformung zu einer gewinnbringenden Struktur führt (Tab. 1, Turn 5). Wie in Abschn. 2.1 dargestellt, gehören zum Planen insbesondere auch Evaluationen hinsichtlich verschiedener Kriterien wie Effizienz, Ästhetik, Fehleranfälligkeit oder Verallgemeinerbarkeit, wie: „Ich empfehle wegen den irrationalen Parametern die Auflösungsformel.“ (weitere Beispiele Tab. 1, Turn 11 und 13).

In einer Voranalyse dieser Codierung zeigten sich Schwierigkeiten beim Codieren von längeren Turns von Lehrpersonen, welche auf verschiedene Prozesse beim Lösen von Gleichungen eingingen, und weiter von Turns ohne expliziten mathematischen Gehalt, wie zum Beispiel „Mhm.“. Darum wurde die Codierung mit zwei zusätzlichen Codes ergänzt: KOMB und CONTIN. Der Code KOMB für Kombinationen beschreibt Turns mit verschiedenen, unzusammenhängend thematisierten Gleichungslöseprozessen, bspw. in der Form von längeren Erklärungen. In solchen Äußerungen wird bspw. sowohl das Ausführen eines Rechnungsschritts thematisiert als auch die Struktur eines anderen Terms. Zudem wurde dieser Code auch für kurze, unspezifische Turns vergeben, welche sich allgemein auf das Gleichungslösen beziehen, wie „Was könnt ihr mir zu dieser Formel erzählen?“ (vgl. Tab. 1, Turn 10). Wenn sowohl Strukturierungsprozesse als auch Ausführungsprozesse thematisiert werden, grenzt sich KOMB von PLAN ab, indem beim Planen Strukturierungsprozesse und Ausführungsprozesse aufeinander bezogen werden.

Der Code CONTIN steht für Continuer und beschreibt Turns ohne explizit thematisierten Gleichungslöseprozess. Solche Turns dienen dem Aufrechterhalten oder Steuern des Gesprächs (vgl. Hugener et al. 2006; Futter 2017). „Mhm.“, „Entschuldigen Sie, man sieht es nicht.“ oder „Einverstanden? “ wären solche Turns (vgl. Tab. 1, Turn 4 und 12). Jeder als CONTIN codierte Turn steht innerhalb einer Sequenz zum Lösen von Gleichungen. Continuer können den Wissensaufbau fördern, wenn danach mathematisches Denken explizit wird, bspw. „Kann das jemand mit seinen eigenen Worten sagen?“ oder „Darf ich hier noch etwas ergänzen?“.

Die beschriebenen Codes STRUKT, AUSF, PLAN, KOMB und CONTIN erlauben eine disjunkte Codierung der einzelnen Turns beim Lösen von Gleichungen. Die Nicht-Codierung eines Turns (NONGL) bedeutet, dass der betreffende Turn außerhalb einer Sequenz zum regelbasierten Lösen von Gleichungen steht.

Äußerungen im Klassengespräch sind oft mehrdeutig, unabgeschlossen, stellen in Frage oder entstehen als subjektive Zugänge zu mathematischen Sachverhalten. Aus sozialkonstruktivistischer Perspektive sind die beschriebenen Äußerungen Elemente des Aushandelns des Gleichungslösens (Vygotsky 1978). Ein Sprechen über die Struktur einer Gleichung entspricht bspw. einem Aushandeln von Strukturierungsprozessen. Inwiefern diese Äußerung wahr ist, spielt eine untergeordnete Rolle, da fehlerbehaftete Aussagen im Gespräch berichtigt werden können.

Die Interraterreliabilität der Codierung bezieht sich auf 20 % des Materials, welches von zwei Ratern mit gymnasialer Lehrerfahrung codiert wurden. Für die sechs disjunkten Codes (STRUKT, AUSF, PLAN, KOMB, CONTIN, NONGL) ergibt sich ein gutes Cohens κ von 0,74.

#### Die Codierung dreier Besprechungsarten von Gleichungen

Neben den thematisierten Gleichungslöseprozessen, welche anhand des Problemlösens entwickelt wurden, sind zusätzlich drei Besprechungsarten unterschieden worden. Diese drei Besprechungsarten orientieren sich am diskursiven Umgang mit multiplen Lösungswegen von Gleichungen: 1. Besprechung einer Gleichung anhand multipler Lösungswege mit einem Vergleich (Mlt_mVgl). 2. Besprechung einer Gleichung anhand multipler Lösungswege ohne einen Vergleich (Mlt_oVgl). 3. Besprechung einer Gleichung ohne multiple Lösungswege, also mit nur einem Weg (Non_Mlt). Durch diese Unterscheidung schließen sich die Besprechungsarten gegenseitig aus.

Eine Gleichung wurde „mit multiplen Lösungswegen besprochen“ codiert, wenn mindestens zwei für alle (schriftlich) sichtbare Lösungswege auftraten. Damit eine Besprechung zusätzlich als Vergleich von multiplen Lösungswegen codiert wurde, musste sinngemäß mindestens eine der folgenden Fragen gestellt werden (entsprechend den Vergleichsaufgaben von Rittle-Johnson und Star 2007):

- Welcher Lösungsweg ist einfacher? Warum gelingt dieser Lösungsweg hier? Welcher funktioniert immer?
- Welche Vor- und Nachteile siehst du in den Lösungswegen? Wie unterscheiden sie sich?
- Erfinde eine Gleichung, bei der du den ersten bzw. zweiten Lösungsweg wählen würdest.

Zur Abgrenzung der Besprechungsarten multipler Lösungswege mit bzw. ohne Vergleich wurde festgelegt, dass jeweils die gesamte Sequenz zu einer Gleichung mit demselben Code identifiziert werden soll. Die Sequenzen des Merkmals Mlt_mVgl und Mlt_oVgl wurden identifiziert und den entsprechenden Turns zugewiesen. Der Code Non_Mlt wurde allen anderen Turns zum regelbasierten Gleichungslösen zugeteilt. Da eine Gleichung meist über mehrere Turns hinweg besprochen wird und da in einem einzelnen Turn nicht explizit werden muss, ob die Gleichung multipel gelöst oder verglichen wird, ist die Codierung der einzelnen Turns nicht unabhängig voneinander. Dies führt zu einer hohen Interraterreliabilität (Cohens κ = 0,998). Die Zuordnung der Codes der Besprechungsarten lässt sich in Tab. 2 anhand von Beispielen nachvollziehen.

**Table 2:** 

	Beschreibung	Beispielsequenzen von Klassengesprächen
Mlt_mVgl	Multiple Lösungswege mit Vergleich	– Eine Vergleichsaufgabe wird besprochen. – Nachdem individuell eine Gleichung gelöst wird, notieren mehrere Lernende ihre Lösungswege an die Wandtafel. Im Anschluss werden die Vor- und Nachteile der Lösungswege gegeneinander abgewogen
Mlt_oVgl	Multiple Lösungswege ohne Vergleich	– Nachdem individuell eine Gleichung gelöst wird, sammelt die Lehrperson verschiedene Lösungswege an der Tafel, ohne sie zu vergleichen. – Nachdem eine Gleichung gemeinsam besprochen wurde, wird gefragt, ob auch ein zweiter Weg, der an die Tafel notiert wird, korrekt sei. Die Wege werden nicht aufeinander bezogen
Non_Mlt	Restliche Turns zum Lösen von Gleichungen	– Eine Gleichung wird gemeinsam in der Klasse besprochen bzw. gelöst. – Eine Lösungsformel der quadratischen Gleichung wird hergeleitet

### Statistische Analysen

Ob Planungsprozesse durch das Vergleichen von multiplen Lösungswegen häufiger besprochen werden, wird gemäß Hypothese (i) zuerst klassenübergreifend mit einer binär logistischen Regression untersucht (Abschn. 3.3.1) und danach gemäß Hypothese (ii) klassenspezifisch mit einem t‑Test für paarweise verbundene Stichproben (Abschn. 3.3.2). Grundlage beider Analysen bildet die Codierung der thematisierten Gleichungslöseprozesse im Klassengespräch und der drei Besprechungsarten von Gleichungen.

#### Klassenübergreifende Analyse der Gleichungslöseprozesse in Abhängigkeit der Besprechungsart

Mit einer binär logistischen Regression lässt sich der Zusammenhang zwischen einer unabhängigen Variablen und einer binär codierten abhängigen Variablen abschätzen (vgl. Backhaus et al. 2018; Urban und Mayerl 2018). Genauer gesagt wird ermittelt, inwieweit eine unabhängige Variable einen Einfluss auf die Wahrscheinlichkeit hat, dass die abhängige Variable eintritt oder nicht. Vorliegend werden demnach Turns zum Gleichungslösen daraufhin analysiert, inwieweit die Besprechungsart die Wahrscheinlichkeit beeinflusst, dass ein spezifischer Gleichungslöseprozess in einem Turn thematisiert wird oder nicht. In jeweils einer binär logistischen Regression wird der Einfluss der unabhängigen, dreigestuften Variablen (Non_Mlt (= 0), Mlt_oVgl (= 1) oder Mlt_mVgl (= 2)) auf eine der drei abhängigen, binär codierten Variablen der Gleichungslöseprozesse (PLAN, STRUKT oder AUSF) ermittelt. Der Einfluss wird als Koeffizient *Exp(B)* ausgewiesen, der sich als Faktor interpretieren lässt, wie sich das Chancenverhältnis (engl. *odds ratio*), ob ein bestimmter Gleichungslöseprozess thematisiert wird oder nicht, in Abhängigkeit von einer Besprechungsart zu einer anderen verändert. Eine Kategorie wird dafür als Referenzkategorie festgelegt (*Odds*_*Kategorie 1*_ _*oder 2*_ *=* Exp(B)*Odds_*Referenzkategorie 0*_), zum Beispiel Non_Mlt. Ein Faktor *Exp(B) *grösser als 1 entspricht einer Erhöhung, ein Faktor kleiner als 1 einer Verringerung der Chancenwahrscheinlichkeit im Vergleich zur Referenzkategorie (Best und Wolf 2012). Ein Modell zu einem Gleichungslöseprozess berechnet dadurch zwei Koeffizienten *Exp(B)*, die den Einfluss einer Referenzkategorie zu den beiden anderen Kategorien bestimmt. Um auch die beiden anderen Kategorien (1 und 2) direkt miteinander zu vergleichen, wird pro Gleichungslöseprozess ein zusätzliches Modell gerechnet, indem eine zweite Referenzkategorie gesetzt wird. Die beiden Modelle dienen dem besseren Vergleich der Kategorien, rechnerisch werden dieselben Zahlen genutzt und Schätzungen vorgenommen, nur die Referenzkategorie ändert sich. Im Modell a wird die Referenzkategorie Non_Mlt = 0 gesetzt und mit den Merkmalsausprägungen Mlt_mVgl bzw. Mlt_oVgl verglichen. Im Modell b wird die Referenzkategorie Mlt_mVgl = 0 gesetzt. So ergeben sich gesamthaft 6 Modelle, nämlich zwei Modelle für die drei Gleichungslöseprozesse (vgl. Tab. 5 und 6 und 7).^3^ Zur Vermeidung einer α‑Fehler-Kumulation der verschiedenen Modellrechnungen wird eine konservative Bonferroni-Korrektur vorgenommen (Holm 1979). In dieser Methode wird das Signifikanzniveau durch die Anzahl Modelle geteilt.^4^ Wichtig zur Einordnung des Verfahrens ist einerseits, dass die Turns nicht unabhängig voneinander codiert sein müssen (vgl. Abschn. 3.2.3). Andererseits werden mit diesem Analyseverfahren alle Turns klassenübergreifend gleichgestellt. Dies erlaubt, Turns als mögliche Lerngelegenheit mit Bezug auf einen Gleichungslöseprozess zu deuten.

#### Klassenspezifische Analyse der Gleichungslöseprozesse in Abhängigkeit der Besprechnungsart

Die klassenspezifischen Auswirkungen der Besprechungsart auf die Gleichungslöseprozesse werden mit einem t‑Test für paarweise verbundene Stichproben betrachtet. Die Vergleichbarkeit der Klassen (mit verschieden vielen Turns pro Besprechungsart) wird durch relative Werte der thematisierten Gleichungslöseprozesse pro Klasse hergestellt. Der t‑Test bestimmt dann die Mittelwertdifferenz der relativen Werte von je zwei Besprechungsarten. So lässt sich testen, ob durchschnittlich in einer Klasse ein bestimmter Gleichungslöseprozess in Abhängigkeit der Besprechungsart häufiger auftritt.

Die paarweise verbundenen Werte entsprechen den relativen Werten der verschiedenen Besprechungsarten. Dadurch wird die Stichprobengröße verkleinert: Klassen mit nur einer Besprechungsart können bspw. nicht berücksichtigt werden. Denn für einen Vergleich der Besprechungsarten einer Klasse müssen mindestens zwei Besprechungsarten vorhanden sein.

Im Vergleich von sich gegenseitig beeinflussenden relativen Werten unter 0,2 bzw. über 0,8 (wie die der Gleichungslöseprozesse) wird nach Warton und Hui (2011) eine logistische Transformation empfohlen.

Die Gegenüberstellung der Besprechungsarten hinsichtlich der drei Gleichungslöseprozesse ergibt für die t‑Tests pro Gleichungslöseprozess drei (vgl. Tab. 9), gesamthaft also neun Modelle. Entsprechend der binär logistischen Regression wird eine Bonferroni-Korrektur vorgenommen.

## Ergebnisse

### Deskriptive Ergebnisse

Die durchschnittliche Aufnahmedauer pro Lektion betrug für die 172 Lektionen circa 38 min (*s* = 5,84). In diesen Lektionen wurden 452 Klassengesprächssequenzen identifiziert, welche etwa zwei Drittel der Gesamtdauer der Lektionen einnehmen. Von den transkribierten Klassengesprächen thematisieren 17.409 von 19.093 Turns das Gleichungslösen. Weil es sich bei den vorliegenden Daten um eine Gelegenheitsstichprobe handelt, sei erwähnt, dass multiple Lösungswege vor allem in den Experimentalgruppen auftraten. Genauer stammen 99,8 % der Turns zu Mlt_mVgl bzw. 83,3 % der Turns zu Mlt_oVgl aus den Experimentalgruppen. Vergleiche bzw. multiple Lösungswege wurden demnach in der Kontrollgruppe kaum bzw. seltener besprochen.

Tab. 3 und 4 zeigen deskriptiv die Verteilung der Codes der Gleichungslöseprozesse und der Besprechungsarten als Kreuztabelle. Tab. 3 bildet die absoluten Zähldaten ab. Aufgrund der disjunkten Codierung entspricht eine Einheit genau einem Code bzw. genau einem Turn. Tab. 4 zeigt ergänzend die relativen Werte. Knapp ein Drittel der Turns wurde beim Lösen von Gleichungen mit multiplen Lösungswegen geäußert (Mlt_oVgl und Mlt_mVgl zusammen). Über drei Viertel der Turns zu multiplen Lösungswegen sind innerhalb von Sequenzen zu Vergleichen. In allen Besprechungsarten wurden Ausführungsprozesse am häufigsten thematisiert (mit circa der Hälfte der Turns). Am zweithäufigsten waren Turns ohne expliziten Gehalt (Continuer). Das restliche Viertel verteilte sich auf PLAN (9 %), STRUKT (12 %) und KOMB (6 %).

**Table 3:** 

*n* = 17.409	PLAN	STRUKT	AUSF	KOMB	CONTIN	Total
Non_Mlt	811	1681	5499	679	3119	11.789
Mlt_oVgl	86	65	693	65	310	1219
Mlt_mVgl	620	329	2099	226	1127	4401
Total	1517	2075	8291	970	4556	17.409

**Table 4:** 

*n* = 17.409	PLAN (%)	STRUKT (%)	AUSF (%)	KOMB (%)	CONTIN (%)	Total (%)
Non_Mlt	7	14	47	6	26	100
Mlt_oVgl	7	5	57	5	25	100
Mlt_mVgl	14	7	48	5	26	100
Total	9	12	48	6	26	100

Ein Vergleich der Zeilen von Tab. 4 lässt vermuten, inwiefern bestimmte Gleichungslöseprozesse durch die Besprechungsart häufiger thematisiert wurden. Außerdem zeigt sich, dass alle Gleichungslöseprozesse in allen Besprechungsarten auftraten und dass die Kategorienanteile von KOMB und CONTIN nahezu konstant verteilt sind. Dies stützt das Vorgehen, die statistischen Analysen (Abschn. 4.2 und 4.3) auf die Unterschiede der drei Gleichungslöseprozesse STRUKT, AUSF und PLAN einzuschränken.

### Klassenübergreifende Ergebnisse der Analyse der Gleichungslöseprozesse

Die binär logistische Regression vergleicht klassenübergreifend die Wahrscheinlichkeiten für das Auftreten eines Gleichungslöseprozesses in jeweils zwei Besprechungsarten. Die Tab. 5, 6 und 7 weisen die binär logistischen Regressionen für jeden Gleichungslöseprozess aus. Da jeweils nur zwei von drei Besprechungsarten miteinander verglichen werden (Non_Mlt, Mlt_oVgl, Mlt_mVgl), wurden pro Gleichungslöseprozess zwei Modelle a und b mit wechselnder Referenzkategorie gerechnet. Der Koeffizient *Exp(B)* entspricht dem Verhältnis der Chancenwahrscheinlichkeiten der verglichenen Kategorien. Mit der Bonferroni-Korrektur sinkt das Signifikanzniveau auf 0,008.

**Table 5:** 

	Modell 1a			Modell 1b		
	*B*	*p*	*Exp*(*B*)	*B*	*p*	*Exp*(*B*)
*Ref.: Non_Mlt*						
Mlt_mVgl	0,797	0,000	2,220	–	–	–
Mlt_oVgl	0,027	0,818	1,027	–	–	–
*Ref.: Mlt_mVgl*						
Non_Mlt	–	–	–	−0,797	0,000	0,450
Mlt_oVgl	–	–	–	−0,770	0,000	0,463
Nagelkerke Pseudo *R*^2^	0,025			0,025		
Effektstärke *f*	0,160			0,160

**Table 6:** 

	Modell 2a			Modell 2b		
	*B*	*p*	*Exp*(*B*)	*B*	*p*	*Exp*(*B*)
*Ref.: Non_Mlt*						
Mlt_mVgl	−0,722	0,000	0,486	–	–	–
Mlt_oVgl	−1,083	0,000	0,339	–	–	–
*Ref.: Mlt_mVgl*						
Non_Mlt	–	–	–	0,722	0,000	2,059
Mlt_oVgl	–	–	–	−0,361	0,010	0,697
Nagelkerke Pseudo *R*^2^	0,024			0,024		
Effektstärke *f*	0,157			0,157

**Table 7:** 

	Modell 3a			Modell 3b		
	*B*	*p*	*Exp*(*B*)	*B*	*p*	*Exp*(*B*)
*Ref.: Non_Mlt*						
Mlt_mVgl	0,042	0,234	1,043	–	–	–
Mlt_oVgl	0,410	0,000	1,507	–	–	–
*Ref.: Mlt_mVgl*						
Non_Mlt	–	–	–	−0,042	0,234	0,959
Mlt_oVgl	–	–	–	0,368	0,000	1,445
Nagelkerke Pseudo *R*^2^	0,004			0,004		
Effektstärke *f*	0,063			0,063		

Die Wahrscheinlichkeit, dass ein Turn einen Planungsprozess thematisiert, war unter der Bedingung Mlt_mVgl im Vergleich zur Bedingung Non_Mlt signifikant erhöht (*Exp(B)* = 2,220; *p* = 0,000). Mit Blick auf Tab. 4 lässt sich diese Erhöhung als eine Verdoppelung der Wahrscheinlichkeit lesen (von 7 auf 14 %). Die Verdoppelung lässt sich jedoch nicht auf die multiplen Lösungswege zurückführen. Denn die Gegenüberstellung von Mlt_oVgl zu Mlt_mVgl verweist auf eine signifikante Verringerung der Planungsprozesse, wenn beim Besprechen der Lösungswege kein Vergleich vorgenommen wird (*Exp(B)* = 0,463; *p* = 0,000). Damit stützen die Daten der binär logistischen Regression die klassenübergreifende Hypothese (i), dass beim Vergleichen von Lösungswegen über alle Äußerungen hinweg häufiger geplant wird. Das vorsichtig zu interpretierende Nagelkerke Pseudo *R*^*2*^ (Best und Wolf 2010) illustriert, dass nicht nur die unabhängige Variable den thematisierten Gleichungslöseprozess eines Turns vorhersagt. Da sich die beiden Modelle a und b jeweils nur hinsichtlich der Referenzkategorie unterscheiden, ergeben sich dieselben Likelihoodschätzungen und darum für beide Modelle dasselbe Nagelkerke *R*^2^. Mit dem Nagelkerke *R*^2^ lässt sich nach Cohen (1988) eine schwache bis mittlere Effektstärke *f* von 0,16 berechnen.

Beim Besprechen von multiplen Lösungswegen wurde das Strukturieren seltener thematisiert (Mlt_mVgl: *Exp(B)* = 0,486, *p* *=* 0,000; Mlt_oVgl: *Exp(B)* = 0,339, *p* *=* 0,000). Unter den Bedingungen mit multiplen Lösungswegen wurde ohne Vergleiche seltener strukturiert (*Exp(B)* = 0,697). Mit der Bonferroni-Korrektur ist der letzte Faktor nicht mehr signifikant (*p* *=* 0,010). Unter der Bedingung Mlt_oVgl wurde typischerweise das Ausführen häufiger besprochen (Non_Mlt: *Exp(B)* = 1,507; Mlt_mVgl: *Exp(B)* = 1,445). Der signifikante Faktor wird aber durch das Nagelkerke *R*^*2*^ von 0,04 und einer niedrigen Effektstärke von 0,063 relativiert.

Neben der Annahme der Hypothese (i) lässt sich somit ein häufigeres Strukturieren beim Lösen ohne multiple Lösungswege und ein häufigeres Ausführen beim Lösen mit multiplen Lösungswegen ohne Vergleich festhalten.

### Klassenspezifische Ergebnisse der Analyse der Gleichungslöseprozesse

Die t‑Tests für paarweise verbundene Stichproben vergleichen die Klassenmittelwerte der relativen Häufigkeiten der drei Gleichungslöseprozesse zwischen den Besprechungsarten. Tab. 8 bietet einen Überblick über die relativen Klassendaten.

**Table 8:** 

*n* = 43	PLAN		STRUKT		AUSF	
Besprechungsart	*m*	*s*	*m*	*s*	*m*	*s*
Non_Mlt	0,073	0,062	0,105	0,072	0,385	0,132
Mlt_oVgl	0,064	0,050	0,060	0,084	0,537	0,197
Mlt_mVgl	0,158	0,096	0,076	0,057	0,423	0,159

Ein Wert von 0,073 unter der Bedingung Non_Mlt und PLAN bedeutet bspw., dass Klassen beim Gleichungslösen mit einem Lösungsweg im Mittel mit dieser Häufigkeit Turns zum Besprechen von Planungsprozessen nutzten. Die Klassendaten weisen große Ähnlichkeit mit den relativen Daten über alle Klassen auf (vgl. Tab. 4): So unterscheiden sich bspw. die Daten zu den Planungs- und Strukturierungsprozessen um höchstens drei bis vier Prozentpunkte. Das heißt, dass die Prozessanteile pro Besprechungsart durch die Klassen ausgemittelt werden. Zusätzlich zeigen die Standardabweichungen in Tab. 8 die vorherrschende Streuung. Das Wertepaar beim Planen von Non_Mlt (*m* = 0,062, *s* = 0,073) illustriert bspw., dass einige Klassen Planungsprozesse nicht besprachen bzw. auch sehr viel häufiger.

Tab. 9 weist alle t‑Tests für paarweise verbundene Stichproben aus. Es werden die Klassenmittelwerte der relativen Daten der drei thematisierten Gleichungslöseprozesse unter jeweils zwei der drei Besprechungsarten miteinander verglichen.^5^

**Table 9:** 

	*m*	*s*	*t*	*df*	*p*	*r (Pearson)*
*Planen (PLAN)*						
Mlt_mVgl – Non_Mlt	0,08	0,12	3,203	25	0,004	0,54
Mlt_oVgl – Non_Mlt	0,01	0,04	0,596	19	0,558	0,14
Mlt_oVgl – Mlt_mVgl	−0,07	0,12	−1,696	9	0,124	0,49
*Strukturieren (STRUKT)*						
Mlt_mVgl – Non_Mlt	−0,02	0,08	−1,226	25	0,232	0,24
Mlt_oVgl – Non_Mlt	−0,06	0,09	−2,856	19	0,010	0,55
Mlt_oVgl – Mlt_mVgl	−0,01	0,13	−0,320	9	0,756	0,11
*Ausführen (AUSF)*						
Mlt_mVgl – Non_Mlt	0,05	0,21	1,206	25	0,239	0,23
Mlt_oVgl – Non_Mlt	0,11	0,21	2,357	19	0,029	0,48
Mlt_oVgl – Mlt_mVgl	0,14	0,22	1,986	9	0,078	0,55

Die Freiheitsgrade dokumentieren den Gebrauch der verschiedenen Besprechungsarten in den Klassen. Die 25 Freiheitsgrade (*df* = 25) in Tab. 9 bedeuten, dass in 26 von 43 Klassen Gleichungen sowohl mit nur einem Lösungsweg als auch mit einem Vergleich multipler Lösungswege besprochen wurden. Multiple Lösungswege ohne Vergleich traten in weniger Klassen auf (vgl. *df* = 19 bzw. *df* = 9). Dass insbesondere nur 10 Klassen multiple Lösungswege sowohl mit als auch ohne Vergleich nutzten, führt zu einer geringeren statistischen Aussagekraft. Zusätzlich bleibt zu bemerken, dass 7 Klassen im t‑Test nicht berücksichtig werden, weil sie nie multiple Lösungswege bei der Besprechung nutzten.

Der t‑Test zeigt, dass Planungsprozesse beim Vergleichen mit circa 7 bis 8 Prozentpunkten häufiger besprochen wurden. Dem entspricht die Verdopplung der klassenübergreifenden Analyse. Diese Unterschiede beziehen sich im t‑Test nur auf diejenigen Klassen, die die entsprechenden Besprechungsarten genutzt haben. Mit der Bonferroni-Korrektur wird das Signifikanzniveau von 0,05 durch die Anzahl Modelle (hier: 9) auf 0,0056 gesenkt. Dadurch ist nur der Unterschied des Planens zwischen dem Vergleichen und den nichtmultiplen Lösungswegen signifikant (*p* = 0,004). Dieser Unterschied lässt sich mit Cohen (1988) als stark bezeichnen (*r* > 0,5). Der Unterschied der 7 Prozentpunkte zwischen den multiplen Lösungswegen mit und ohne Vergleich ist aufgrund der Freiheitsgrade nicht mehr signifikant. Damit stützen die Daten der t‑Tests für paarweise verbundene Stichproben die klassenspezifische Hypothese (ii), dass beim Vergleichen von Lösungswegen in einer Klasse häufiger Planungsprozesse besprochen werden – mindestens im Kontrast zum Lösen mit nur einem Lösungsweg. Da die anderen t‑Tests nicht signifikant sind, wird darauf verzichtet, genauer auf die Mittelwertunterschiede einzugehen.

## Diskussion

### Zusammenfassung und Implikationen

Im Rahmen dieses Beitrags wurden Klassengespräche zum Lösen von Gleichungen analysiert. Sowohl die klassenübergreifende Hypothese (i) als auch die klassenspezifische Hypothese (ii), dass beim Besprechen von multiplen Lösungswegen mit Vergleichen Planungsprozesse häufiger auftreten, können durch die vorliegende Untersuchung gestützt werden, insbesondere, wenn gegen das Besprechen ohne multiple Lösungswege kontrastiert wird. Bei der klassenspezifischen Hypothese (ii) war der Unterschied zwischen Klassengesprächen von multiplen Lösungswegen mit vs. ohne Vergleich jedoch aufgrund des reduzierten Samples nicht signifikant. Beide Analysen zeigen, dass Planungsprozesse beim Vergleichen etwa doppelt so oft besprochen wurden. Diese Häufung der Thematisierung von Planungsprozessen könnte die Lernwirksamkeit von Vergleichen zum Beispiel in Durkin et al. (2017) oder Durkin et al. (2021) erklären. Das Vergleichen im Klassengespräch ist nicht eine lernförderliche Black Box, sondern führt zu einer vermehrten Thematisierung von Planungsprozessen, was sich wiederum auf die Flexibilität der Lernenden auswirken könnte.

Kooloos et al. (2020) haben aufgezeigt, dass das Besprechen von mehreren Lösungswegen im Mathematikunterricht die Diskussionskultur verändert. Auch der vorliegende Artikel zeigt klassenübergreifend und teilweise klassenspezifisch, dass sich beim Vergleichen von multiplen Lösungswegen die besprochenen Inhalte verändern. Fragen zu den Gelingensbedingungen von Verfahren, zu Vor- und Nachteilen der Lösungswege oder zur Evaluation von Lösungswegen zum Beispiel hinsichtlich der Effizienz können als Möglichkeiten gesehen werden, Planungsprozesse zu besprechen. Beim Besprechen multipler Lösungswege ohne Vergleich traten Planungsprozesse nicht häufiger auf als beim Besprechen nur eines Lösungswegs. Ohne den Vergleich der Lösungswege im Klassengespräch blieb der Fokus vor allem beim korrekten Ausführen der Umformungen. Dies deutet darauf hin, dass einerseits Lösungswege nicht immer evaluiert werden, und ergänzt die Ergebnisse von Krug und Schukajlow (2020) zur Entwicklung prozeduraler Metakognition durch multiple Lösungswege mit der Bedeutung der Vergleiche beim Nutzen multipler Lösungswege. Andererseits bieten entsprechende Fragestellungen einen Orientierungspunkt, dass und wie sich Planungsprozesse thematisieren lassen. Dieses Ergebnis lässt sich mit Alfieri et al. (2013) in Verbindung bringen. Die Metastudie zeigt, dass das Vergleichen vor allem dann zu besseren Lernergebnissen führt, wenn gleichzeitig nach Ähnlichkeiten gefragt wird, verallgemeinert wird oder der Vergleichsgegenstand gesehen (bzw. erkannt) werden kann. Vergleichsaufgaben (wie in Abb. 1) erfüllen diese drei Kriterien. Da die Metastudie aber auch Vergleiche zwischen unterschiedlichen Darstellungen miteinbezieht, während die hier vorgestellten Vergleiche in derselben, algebraischen Darstellung vorliegen, sind die Übereinstimmungen mit Alfieri et al. (2013) mit Vorsicht zu betrachten.

Auf einer allgemeineren Ebene zeigen die Ergebnisse, dass in allen unterschiedenen Besprechungsarten alle Gleichungslöseprozesse thematisiert wurden. Mit etwa der Hälfte aller Turns wurden Ausführungsprozesse am häufigsten besprochen. Mit anderen Worten bildeten die verschiedenen Term- und Äquivalenzumformungen das inhaltliche Zentrum des Unterrichts. Dies entspricht den Instruktionsanalysen der OECD-Studie zu quadratischen Gleichungen von Bell et al. (2020, S. 115f.) insofern, als dort die Lernenden ebenfalls häufig Regeln und Verfahren zusammenfassten und anwendeten. Es steht außer Frage, dass Ausführungsprozesse innerhalb einer Unterrichtseinheit wichtig sind, wo das Nutzen einer Auflösungsformel für quadratische Gleichungen, des Klammeransatzes für Trinome oder des quadratischen Ergänzens erlernt und eingeübt werden soll. Die vorliegenden Daten zeigen, dass bei diesen Ausführprozessen aber weniger besprochen wird, warum diese Umformungen (und nicht andere) gemacht werden oder inwiefern die Umformungen mit den gegebenen Gleichungen zusammenhängen. Denn diese Fragen evozieren Planungsprozesse. Die Forderung nach Flexibilität beim Lösen von Gleichungen bedeutet letztlich, dass Lernende Antworten auf die Frage finden, wann eine (bestimmte) Umformung zielführend ist. Darum sind Planungsprozesse die Grundlage für flexibles Gleichungslösen. Die vorliegenden Daten zeigen, dass solche Planungsprozesse prinzipiell auch ohne das Vergleichen von Lösungswegen thematisiert werden können, Vergleiche aber helfen können, Planungsprozesse explizit werden zu lassen.

Ein Viertel der Turns zum Gleichungslösen war mathematisch nicht explizit (CONTIN) und bestand meist aus wenigen Wörtern (bspw. „Ja. Mhm. Genau.“). Die kommunikative Funktion dieser Turns ist nicht gering zu schätzen, weil um das gegenseitige Verständnis geht („Mhm.“,„Ja. Das habe ich verstanden.“, „Ist das so in Ordnung?“) oder auch die Orchestrierung des Klassengesprächs („Wer kann das mit seinen eigenen Worten erklären?“, „Kann jemand noch etwas dazu sagen?“; vgl. z. B. Howe et al. 2019; Michaels et al. 2010; Pauli und Reusser 2018). Mathematische Inhalte werden in diesen Turns jedoch nicht ausgedrückt. Neben der Konstanz der Häufigkeit dieser Continuer in den drei Besprechungsarten fällt auch die Konstanz der Turns mit verschiedenen thematisierten Gleichungslöseprozessen (KOMB) auf. Solche Turns waren eher selten (5 %, vgl. Tab. 4) und wurden typischerweise von einer Lehrperson geäußert. Eine Ausnahme bildete bspw. eine Präsentation eines Lösungsweges von Seiten der Lernenden. Gemeinsam ist diesen Turns, dass sich Personen länger äußern.

Zwei weitere signifikante Unterschiede der klassenübergreifenden Verteilungen der Gleichungslöseprozesse sollen kurz diskutiert werden. Erstens wurde ein höherer Anteil von besprochenen Strukturierungsprozessen beim Lösen von Gleichungen ohne multiple Lösungswege berichtet. Eine illustrierende Unterrichtssituation wäre die Einführung von quadratischen Gleichungen, wo bspw. eine quadratische Gleichung an die Wandtafel geschrieben und allgemein definiert wurde. Das führt zu einer Besprechung von Strukturierungsprozessen. Zweitens wurden beim Lösen mit multiplen Lösungswegen ohne Vergleich Ausführungsprozesse vermehrt besprochen. Eine typische Situation wäre, wenn ein Lernender im Klassengespräch wissen will, ob sein persönlicher Lösungsweg ebenfalls korrekt ist. Eine solche Unterrichtssituation fokussiert auf die Ausführungsprozesse. Spezifische Besprechungssituationen nehmen also auch spezifisch auf die Gleichungslöseprozesse Bezug.

Verschiedene Studien berichteten bislang über Probleme bei der erfolgreichen Umsetzung von multiplen Lösungswegen im Unterricht (z. B. Silver et al. 2005; Lynch und Star 2014; Star et al. 2015). Auch die vorliegenden Daten weisen auf Schwierigkeiten beim Nutzen von Vergleichen hin. Dies lässt sich mit Hilfe der klassenspezifischen Analyse zeigen. So lässt sich bspw. aus den Klassendaten (Tab. 8) entnehmen, dass beim Vergleichen im Mittel 15,8 % der Turns Planungsprozesse thematisierten – bei einer Standardabweichung von 9,6 Prozentpunkten. Das heißt, dass mit Vergleichsaufgaben und zugehörigen Fragestellungen (wie in Abb. 1) einige Klassen Planungsprozesse sehr viel häufiger bzw. sehr viel seltener besprochen haben. Dazu passen die entsprechenden Klassenwerte beim Besprechen ohne multiple Lösungswege (*m* = 0,073, *s* = 0,062). Die hohen Standardabweichungen weisen aber auf vereinzelte Klassen hin, welche das Planen nicht häufiger thematisierten. Folglich kommt den einzelnen Klassengesprächen eine entscheidende Rolle dafür zu, was thematisiert wird. Gleichungslösen wird im Schulunterricht der Sekundarstufen 1 und 2 oft interpretiert als das Ausüben von Routineaufgaben (Stiller et al. 2021). Seltenes Thematisieren von Planungsprozessen könnte darum darauf hindeuten, dass im analysierten Unterricht das Gleichungslösen in erster Linie auf diese Routine ausgelegt ist und nicht auf das Problemlösen. Dass beim Vergleichen von Lösungswegen Planungsprozesse häufiger besprochen werden, könnte demnach (paradoxerweise) eine Ursache dafür sein, wenn Vergleiche im Unterricht wenig genutzt werden. Hier stellt sich die grundlegende mathematikdidaktische Frage, wozu der Algebraunterricht dienen soll. Nach Hefendehl-Hebeker und Rezat (2015, S. 142) sollen Schülerinnen und Schüler in der Algebra nicht nur Umformungsautomatismen ausführen, sondern auch die formale Sprache und deren Sinn und Nutzen verstehen lernen. Die klassenspezifische Analyse deutet darauf hin, inwiefern dies in Anbetracht der Lernziele und Lehrmittel zum Lösen quadratischer Gleichungen für die geführten Klassengespräche interpretiert wurde und werden kann.

### Limitationen

Wie in Abschn. 3.1 beschrieben, handelt es sich bei den untersuchten Videografien um eine Gelegenheitsstichprobe. In Abschn. 4.1 wurde angemerkt, dass das Nutzen multipler Lösungswege vor allem in Klassen stattfand, deren Mathematiklehrperson vorgängig eine Weiterbildung zum Vergleichen von Lösungswegen besucht hatte. Vergleiche von Lösungswegen traten im herkömmlichen Unterricht der Kontrollgruppe nur marginal auf. Der seltene Gebrauch von Vergleichen führte zu einer stark reduzierten Stichprobe bei den paarweise verbundenen t‑Tests: Es ließen sich zum Beispiel nur 10 Klassen (*df* = 9) gegenüberstellen, welche multiple Lösungswege sowohl ohne als auch mit Vergleich genutzt haben. Daraus erklärt sich auch das nichtsignifikante Ergebnis zur klassenspezifischen Hypothese (ii).

Der Problemlöseprozess wird oft als chronologisches Phasenmodell verstanden (vgl. Rott 2014). Im Unterschied dazu können die unterschiedenen Gleichungslöseprozesse im Klassengespräch immer auftreten. Denn mit jeder Äußerung kann das Klassengespräch hinsichtlich der Gleichungslöseprozesse gelenkt werden. Das erschwerte die reliable Codierung der Gleichungslöseprozesse (Cohens κ = 0,74). Eine Verbesserung wäre jedoch schwierig zu bewerkstelligen, da Ausführ- und Strukturierungsprozesse nicht trennscharf sind. Die Strukturierung des Terms (*x* + 1)^2^ ist bspw. davon abhängig, dass sich der Term auch ausmultiplizieren lässt.

Planungsprozesse wurden in diesem Beitrag weit gefasst. Sowohl Turns zum zielorientierten Umformen wie Turns zur Evaluation von Lösungswegen wurden als PLAN codiert. So lässt sich nur vermuten, dass sich das häufigere Planen beim Vergleichen zu einem hohen Anteil auf die Evaluation der Lösungswege zurückführen lässt. Eine detailliertere Codierung der Planungsprozesse im Sinne des Planens, Monitorings und Evaluierens nach Desoete (2008) oder Lucangeli et al. (1998) wäre künftig zu bedenken. Darüber hinaus hätten die verschiedenen Turns zusätzlich qualitativ evaluiert werden können.

Kritisch lässt sich fragen, wie die unterschiedlichen Verteilungen zu bewerten sind, zum Beispiel die Zunahme der thematisierten Planungsprozesse von 7 auf 14 Prozentpunkte vom Klassengespräch ohne multiple Lösungswege zum Klassengespräch mit Vergleich (vgl. Tab. 4). Dazu sind folgende drei Aspekte zu berücksichtigen:

Erstens wird allgemein festgestellt, dass metakognitive Kompetenzen selten thematisiert werden, oft unbewusst bleiben und zu fördern wären (Shilo und Kramarski 2019). Zweitens sei darauf hingewiesen, dass die Höhe der Prozentpunkte der thematisierten Gleichungslöseprozesse sich aufgrund der disjunkten Codierung der Turns konkurrenzieren. Das heißt, wären alle fünf Codes (STRUKT, AUSF, PLAN, KOMB, CONTIN) gleich oft vergeben, wären alle bei 20. Drittens soll betont werden, dass die unterschiedenen Gleichungslöseprozesse nicht dazu dienen sollen, die Planungsprozesse als qualitativ höherwertig aufzufassen: Um Gleichungen lösen zu können, braucht es alle drei Löseprozesse. Diese drei Aspekte ordnen die Höhe der Prozentanteile des Planens ein und unterstreichen die Bedeutung der Verdoppelung des Planens beim Vergleichen – auch im Hinblick auf die Förderung der Flexibilität.

Gleichwohl stellt sich in diesem Kontext die Frage, inwiefern sich Äußerungen von einzelnen Lernenden oder von der Lehrperson auf alle Individuen der Klasse beziehen lassen können, zum Beispiel für die Interpretation des häufigeren Planens.

### Fazit

Der vorliegende Artikel bietet einen fachdidaktischen Zugang, wie Gesprächsanalysen mit curricularen Lernzielen verbunden werden können. Im spezifischen Fall der Förderung des flexiblen Gleichungslösens zeigte sich, dass Planungsprozesse als theoretisch wertvoll und deshalb erwünscht erachtet werden. Zudem treten diese Planungsprozesse in Klassengesprächen zum Vergleichen von multiplen Lösungswegen doppelt so häufig auf. Durch das Auftreten tragen sie zum flexiblen Lösen von algebraischen Gleichungen bei. Damit dies gelingen kann, sind geeignete Aufgaben und Impulse der Lehrperson förderlich, welche im Klassengespräch das Vergleichen und Evaluieren von Lösungswegen und damit von Planungsprozessen anregen und herausfordern.

Dass beim Besprechen von Gleichungen ohne multiple Lösungswege Planungsprozesse seltener besprochen werden, weist darauf hin, dass Gleichungen eher als Routineaufgaben inszeniert werden und nicht als Problemlöseaufgaben. Vergleiche reichern diese Routinen an. Letztlich bestimmt aber die Klasse, worüber gesprochen wird. Darum stellt sich im Rahmen der Lehrerinnen- und Lehrerbildung die mathematikdidaktische Herausforderung, wie die Verbindung der Lernziele mit den zu besprechenden Inhalten im Unterricht angeleitet werden sollte, zum Beispiel mit dem Verdeutlichen des Zwecks von Vergleichsaufgaben oder indem beim Gleichungslösen die Prozesse des Strukturierens, Ausführens und Planens explizit erwähnt werden.

## Footnotes

1 Die Studie wurde vom Schweizerischen Nationalfonds finanziert, Projektnummer 100019_162686/1.

2 Mein tiefster Dank gilt der Studie, deren Finanzierung, den zahlreichen Lehrpersonen und insbesondere den Schülerinnen und Schülern, die Teil dieser Arbeit geworden sind.

3 Die binär logistischen Regressionen lassen sich auch berechnen, indem die Kategorien paarweise und unter Einschränkung der zwei untersuchten Kategorien verglichen werden. Das würde zu zusätzlich auszuweisenden Zahlen führen (*n*, Nagelkerke *R*^*2*^ und Effektstärke *f*) und bräuchte drei statt zwei Modelle pro Gleichungslöseprozess. Eine solche Berechnung ergibt dieselben Resultate und wurde zur Kontrolle getätigt.

4 Zur Vereinfachung werden die verschiedenen Modelle trotz der gerichteten Hypothese gleichbehandelt. Allgemein ist die Frage nach der Anzahl mitzurechnender Modelle nicht abschließend zu beantworten (Perneger 1998).

5 Für relative Daten unter 0,2 oder über 0,8 wird eine Logit-Transformation vorgeschlagen (vgl. Warton und Hui 2011). Die entstehende nichtlineare, logistische Skala verunmöglicht eine kohärente Interpretation des Mittelwertunterschieds. Da sich die Ergebnisse mit bzw. ohne Transformation im Wesentlichen entsprechen, werden die nicht transformierten Daten ausgewiesen. Die Transformation äußert sich in zwei Unterschieden: Erstens erhöht sich die Signifikanz, zweitens verringern sich teilweise die Freiheitsgrade um bis zu 5 Grade, da sich die Werte 0 und 1 nicht logit-transformieren lassen. Dies trat insbesondere bei Planungsprozessen auf, wenn Klassen nie Planungsprozesse besprochen hatten.
